# Permeability Benchmarking: Guidelines for Comparing *in Silico*, *in Vitro*, and *in Vivo* Measurements

**DOI:** 10.1021/acs.jcim.4c01815

**Published:** 2025-01-17

**Authors:** Christian Jorgensen, Raleigh M. Linville, Ian Galea, Edward Lambden, Martin Vögele, Charles Chen, Evan P. Troendle, Fiorella Ruggiu, Martin B. Ulmschneider, Birgit Schiøtt, Christian D. Lorenz

**Affiliations:** † School of Medicine, Pharmacy and Biomedical Sciences, Faculty of Science & Health, University of Portsmouth, Portsmouth PO1 2DT, Hampshire, U.K.; ‡ Dept. of Chemistry, Aarhus University, Langelandsgade, 140 8000 Aarhus C, Denmark; § The Picower Institute for Learning and Memory, Massachusetts Institute of Technology, 43 Vassar Street, Cambridge, Massachusetts 02139, United States; ∥ Clinical Neurosciences, Clinical and Experimental Sciences, Faculty of Medicine, University of Southampton, Southampton SO16 6YD, U.K.; ⊥ Dept. of Chemistry, King’s College London, London WC2R 2LS, U.K.; # Department of Computer Science, Stanford University, Stanford, California 94305, United States; 7 Department of Molecular and Cellular Physiology, Stanford University, Stanford, California 94305, United States; 8 Institute for Computational and Mathematical Engineering, Stanford University, Stanford, California 94305, United States; 9 Synthetic Biology Group, Research Laboratory of Electronics, Massachusetts Institute of Technology, Cambridge, Massachusetts 02139, United States; 10 Wellcome−Wolfson Institute for Experimental Medicine, School of Medicine, Dentistry and Biomedical Sciences, Queen’s University Belfast, Belfast, County Antrim, BT9 7BL, Northern Ireland, U.K.; 11 Kimia Therapeutics, 740 Heinz Avenue, Berkeley, California 94710, United States; 12 Dept. of Engineering, King’s College London, London WC2R 2LS, U.K.

## Abstract

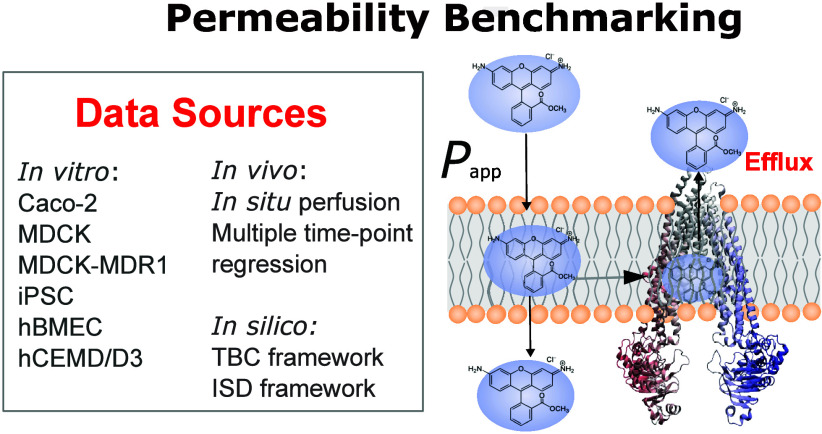

Permeability is a measure of the degree to which cells can transport molecules across biological barriers. Units of permeability are distance per unit time (typically cm/s), where accurate measurements are needed to define drug delivery in homeostasis and to model dysfunction occurring during disease. This perspective offers a set of community-led guidelines to benchmark permeability data across multidisciplinary approaches and different biological contexts. First, we lay out the analytical framework for three methodologies to calculate permeability: *in silico* assays using either transition-based counting or the inhomogeneous-solubility diffusion approaches, *in vitro* permeability assays using cells cultured in 2D or 3D geometries, and *in vivo* assays utilizing *in situ* brain perfusion or multiple time-point regression analysis. Then, we demonstrate a systematic benchmarking of *in silico* to both *in vitro* and *in vivo*, depicting the ways in which each benchmarking is sensitive to the choices of assay design. Finally, we outline seven recommendations for best practices in permeability benchmarking and underscore the significance of tailored permeability assays in driving advancements in drug delivery research and development. Our exploration encompasses a discussion of “generic” and tissue-specific biological barriers, including the blood–brain barrier (BBB), which is a major hurdle for the delivery of therapeutic agents into the brain. By addressing challenges in reconciling simulated data with experimental assays, we aim to provide insights essential for optimizing accuracy and reliability in permeability modeling.

## Introduction

Drug delivery research is a multidisciplinary field aimed at improving the effectiveness and safety of therapeutic interventions. The challenges to get a drug on the market are many and well evidenced elsewhere.^1^ One such challenge is to ensure the safe delivery of the chemical agents to the site of action in the body. As pointed out elsewhere,^2^ between 2000 and 2015, less than 14% of drugs at stage 1 clinical trial went on to receive Food and Drug Administration (FDA) approval,^2,3^ highlighting the scale of the difficulties faced. Consequently, a myriad of precision medicine *in vitro* or *in vivo* approaches have been devised to predict drug delivery. However, routine permeability estimations of therapeutic passage across key biological barriers remain a formidable challenge.^3^

*In silico* computational screening approaches have been developed to overcome such challenges. For central nervous system (CNS) delivery, early stage screening models including Lipinski’s rule of five,^4^ as well the pharmacokinetic predictor *QikProp* program of Schrödinger,^5^ enable the prediction on the probability of a drug crossing membranes based on the chemical properties of a drug. Additionally, emerging artificial intelligence (AI)-based cheminformatic models are working to enhance the predictive capabilities by integrating more complex patterns and interactions.^6^ However, these predictive models are limited in providing detailed insights into the physical process of solutes crossing biological barriers.^7,8^

To address the challenge of assessing barrier penetration beyond scaffold optimization, additional techniques are required. For transport across other membranes, such as in kidney disease therapeutics, physiologically based pharmacokinetic (PBPK) models have proven popular.^9−13^ Similarly, a recent systems biology simulation approach integrates pharmacokinetic models with molecular mechanics techniques to directly incorporate tissue-specific vascular architectures as model inputs.^14^

The use of tissue-specific permeability (*P*) as a precise measure of barrier penetration^15,16^ has proven to be useful in the later stages of drug development.^3,17−20^ It should be noted that permeability can be measured with *in vitro*, *in vivo*, or *in silico* methodologies, each with their own advantages, challenges and limitations. In recent years, a focus on benchmarking simulated permeability values to experimental permeabilities has been carried out extensively,^3,20−26^ with a particular focus on ensuring reproducible values. Despite all this work, a systematic comparison of permeabilities still comes with many caveats and is not a routine procedure. Thus, the purpose of this review article is 2-fold: (1) to provide the theoretical underpinnings of *in silico* permeability and how to benchmark to these findings to *in vitro* and *in vivo* reference data, and (2) to raise understanding on the inherent challenges in utilizing experimental data *as is*, including the inherent statistical variance among different experimental benchmark data sets. The reader is strongly encouraged to use this work as a steppingstone to further examine nuances and underlying assumptions in permeability calculations, beyond the blind use of these data sets.

### Complex Membranes

Cell membranes contain a large variety of lipid types and are crowded with embedded proteins. Their inherent plasticity makes them essential for cell functioning. Complex cellular membranes are characterized by a heterogeneous lateral organization that is poorly understood. When molecules are simulated across complex membranes, the time scales to accurately sample such processes tend to be longer and computationally more demanding.^27^

A prime example of a complex membrane is the plasma cell membrane, which separates the interior of the cell from the outside environment and directly regulates the transport of materials.^27^ The plasma membrane contains hundreds of different types of lipids,^28^ which are organized in a highly heterogeneous fashion. As such, the plasma membrane is implicated in all aspects of endogenous drug delivery. Because a plasma membrane is present across all cells, selective drug delivery has sought to target more specialized membrane barriers, including the blood–brain barrier endothelial membrane^29^ for CNS drug delivery,^30^ and the human *Stratum Corneum* portion of the outer skin for transdermal drug delivery.^31^ These plasma membranes display highly heterogeneous protein machinery across cell types, apical versus basolateral surfaces, and disease states, adding additional complexity for modeling.

The BBB represents a critical interface between the bloodstream and the CNS, regulating the transport of molecules and safeguarding the brain from potentially harmful substances.^32,33^ Dysfunction of the BBB is implicated in the pathogenesis of various neurodegenerative and psychiatric disorders, including Alzheimer’s disease, Parkinson’s disease, and depression.^34^ Thus, understanding the mechanisms underlying molecular transport across the BBB is essential for developing effective treatments for these and other debilitating conditions. While there are approximately 1700 FDA-approved drugs, the brain exposure is only known for around 200 of these compounds.^35,36^ This knowledge gap poses a significant barrier to repurposing existing drugs for CNS disorders, highlighting the critical need for innovative technologies capable of early identification of therapeutics with the potential to penetrate the blood–brain barrier (BBB).^37^

Several experimental techniques were developed to study complex heterogeneous membranes such as the BBB, focusing on the lateral organization of lipids, such as Time-of-Flight Secondary Ion Mass Spectrometry (ToF-SIMS) imaging,^38^ as well as the permeability of solute through membranes, such as transwell assays.

*In silico* methods to investigate membrane properties have advanced rapidly in the past decade. Tools such as FATSLiM,^39^ LiPyphilic,^40^ PyLipID,^41^ MDTraj^42^ and MDAnalysis^43^ enable the examination of membrane properties with atomistic granularity. The lipid diffusion constant and mean-square displacement (MSD),^44^ the membrane curvature and the applied stress,^45^ as well as their dependence on lipid composition, the angular dynamics of embedded membrane proteins,^46^ the area per lipid and bilayer thickness,^47^ are all commonly explored parameters. It is furthermore known that membrane composition affects the permeabilization of biological materials.^48^ For this reason, moving away from model membranes to high-resolution complex membrane compositions is important to get as close to the experimental conditions.

### Experimental Approaches to Vascular Permeability and ADME

The ADME principle, denoting *absorption, distribution, metabolism, and excretion*, summarizes the internal processes that describe how a drug moves throughout and is processed by the body.^49,50^ Distribution describes how a drug moves throughout the body, which is strongly dependent on blood flow, binding to plasma proteins, and the permeability of capillaries. For example, drugs that strongly bind to plasma proteins and/or display low permeability across the BBB will be unable to exert effects within the CNS. A similar concept, bioavailability, denotes the fraction of the originally administered drug that arrives in systemic circulation and depends on the properties of the substance and the mode of administration.^49,50^ Bioavailability plays a crucial role in the functioning of drugs regulated by key tissue barriers, be it intestinal, epithelial, or endothelial, and so forth. Vascular permeability is key to where a drug goes, and thus, what effect a drug has.

Numerous experiments have been conducted to investigate membrane permeability across various drug types. However, these approaches have inherent limitations and are applicable only under specific conditions and for certain drug types, including small molecules, peptides, recombinant proteins, antibodies, and nanoparticles. Several critical parameters have been identified, such as molecular volume, rotatable bonds, polar surface area, and charged groups.^51^ Generally, small, lipid soluble, and noncharged compounds exhibit better permeability to cross cell membranes through passive transport, while charged small molecules can permeate the membranes via active transport. Further simplified *in vitro* systems can mimic the lipid bilayer itself without use of cell culture, including lipid vesicles, supported lipid bilayers, and droplet interface bilayers. These approaches have rapidly advanced and have the advantage of being more directly comparable to *in silico* measurements.^52^ Peptides are sized between small molecules and proteins, and they can traverse cell membranes either via suitable hydrophobicity and a neutral charge or through receptor-mediated transcytosis. Peptide permeation has been studied using both experimental and computational methods,^53,54^ and multiple experimental methods have been widely used in peptide permeation studies, e.g., transwell assay,^55^ liposome permeation assay,^56,57^ electrical conductivity^52,58^ and microfluidic permeation assay.^52,59−61^

In Table 1 we illustrate a range of compound permeabilities for the case of establishing tissue-specific permeability assay, where values are selected from assays that can serve as a proxy or a model for CNS transport. This table showcases the challenging and diverse set of assay types used to estimate these. These challenges will be described in full detail in the section on *Benchmark Challenges*.

**Table 1:** Dataset of Blood-Brain Barrier Active Compounds Ranked by *Decreasing* Permeability Value^a^

Molecule	MW (g mol^–1^)	Log *K*_ow_	*P*_app_ (cm s^–1^)	*P*_app_ Reference	*In vitro* model or *in vivo* method
**Propanol**	60.1	0.05	3.30 × 10^–3^	[Brahm 1983]^62^	RBC
**Ethanol**	46.1	–0.31	1.10 × 10^–3^	[Brahm 1983]^62^	RBC
**Nicotine**	162.2	1.17	1.78 × 10^–4^	[Garberg 2005]^63^	Caco-2/MDCK
**Ketoprofen**	254.3	3.12	8.00 × 10^–5^	[Sun 2002]^64^	Caco-2
**Effexor**	277.0	3.2	6.00 × 10^–5^	[Hellinger 2012]^65^	Caco-2/MDCK
**Bupropion**	239.7	3.60	4.75 × 10^–5^	[Summerfield 2007]^66^	MDCK-MDR1
**Diazepam**	284.7	2.82	4.60 × 10^–5^	[Summerfield 2007]^66^	MDCK-MDR1
**Naproxen**	230.3	3.18	3.90 × 10^–5^	[Pade 1998]^67^	Caco-2
**Clozapine**	326.8	3.23	3.90 × 10^–5^	[Yang 2024]^68^	hCMECD/D3
**Risperdal**	410.4	3.49	3.00 × 10^–5^	[Summerfield 2007]^66^	MDCK-MDR1
**Dilantin**	252.3	2.47	2.70 × 10^–5^	[Summerfield 2007]^66^	MDCK-MDR1
**Ibuprofen**	206.3	3.97	2.70 × 10^–5^	[Wang 2019]^7^	MDCK
**Buspirone**	422.0	1.95	2.50 × 10^–5^	[Boateng 2023]^69^	Caco-2
**Ritalin**	233.1	2.25	2.47 × 10^–5^	[Yang 2016]^70^	MDCK
**Caffeine**	194.2	–0.07	2.10 × 10^–5^	[Wang 2019]^7^	MDCK
**Duloxetine**	297.4	4.00	1.66 × 10^–5^	[Hellinger 2012]^65^	Caco-2/MDCK
**Lacosamide**	250.3	0.73	1.60 × 10^–5^	[Zhang 2013]^71^	Caco-2
**Glycerol**	92.09	–1.8	9.50 × 10^–6^	[Shah 1989]^72^	BMEC
**Ethosuximide**	141.2	0.38	9.00 × 10^–6^	[Summerfield 2007]^66^	MDCK-MDR1
**Sertraline**	306.2	5.10	2.10 × 10^–6^	[Summerfield 2007]^66^	MDCK-MDR1
**Temozolomide**	194.1	0.4	1.86 × 10^–6^	[Avdeef 2012]^73^	Brain perf (3D)
**Atenolol**	266.3	0.16	1.30 × 10^–6^	[Adson 1995]^74^	Caco-2
**Sucrose**	342.3	–3.7	1.00 × 10^–6^	[Franke 1999]^75^	Caco-2
**Nadolol**	309.4	0.81	3.30 × 10^–7^	[Yamashita 2000]^76^	Caco-2
**Doxorubicin**	543.5	1.27	1.00 × 10^–7^	[Hellinger 2012]^65^	Caco-2/MDCK
**Rhodamine 123**	380.8	1.06	0.80 × 10^–7^	[Katt 2019]^77^	iPSC

a The following parameters are supplied: (A) molecular weight (MW; g/mol), (B) octanol–water partition coefficient (Log *K*_ow_), (C) experimental permeability *P*_app_ (cm s^–1^), (D) literature reference, (E) The models or methodologies employed to measure the permeability are red blood cell (RBC), immortalized cell line of human colorectal adenocarcinoma cells (Caco-2), Madin-Darby canine kidney (MDCK), Madin-Darby canine kidney with Multidrug Resistance Protein 1 expressed (MDCK-MDR1), rat brain perfusion (3D), Human Induced pluripotent stem cells (iPSC)-derived cells, and Human Cerebral Microvascular Endothelial Cell Line (hCMEC/D3) mono-culture cells.

#### Permeability Estimations

##### Diffusion versus Solvent Drag Components to Flux of Solutes

Before outlining the theoretical expressions for permeability *in silico*, *in vitro* and *in vivo,* we note that the molecular flux used to describe permeability in *in vitro* experiments has both a diffusive component and a solvent drag component (convective flow), both of which are important. Dynamics in molecular dynamics simulations are typically diffusive, and do not include convective flow in the setup, due to the system size being smaller than those in the experiments. Solvent drag from convective flow in experiments can be a major component to the permeability, and is highly dependent on assay parameters like pressure gradients.^78−81^ This difference in the theoretical formulation of permeability could be one reason for the order of magnitude discrepancy between computational and experimental permeabilities.

### Paracellular and Transcellular Permeability

**Figure 1:**
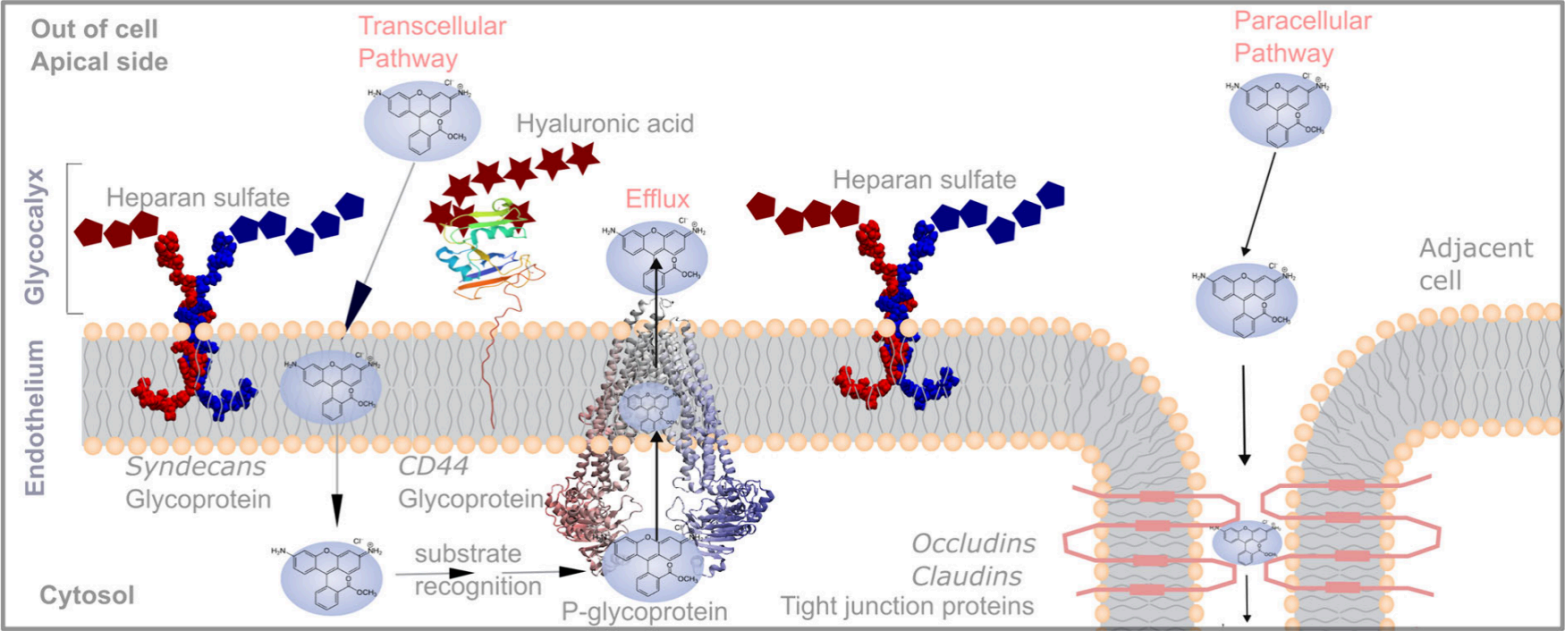
**Transcellular and paracellular pathways across cellular barriers.** Schematic of the apical endothelial cell membrane with embedded glycoproteins (syndecans, CD44, P-gp). The glycocalyx extends beyond the endothelial cell membrane and is made up of the chains of glycoproteins radiating outward, which preorganize the transport of small-molecules across the endothelial cell membrane.^96−98^ Sulfates denoted by ⬠, and hyaluronic acid denotes by ☆. A transcellular permeation event with permeability *P*_transcellular_ for a small molecule is depicted, as well as the substrate recognition for solutes that are P-gp substrates, and their subsequent efflux. The cell–cell tight junction is illustrated, which acts to restrict the paracellular pathway. Note that on the basolateral surface of the endothelial cells there is a specialized protein network termed the basement membrane, which also can be a barrier to permeability.

While most bilayer simulations or model setups *in silico* target solely the transcellular (through-cell) pathway, with associated terminology *P*_transcellular_, the paracellular permeability, with associated terminology *P*_paracellular_ is another major component of *in vitro* and *in vivo* permeability taken by drugs through the extracellular spaces between adjacent cells (Figure 1). In the context of vascular biology, distribution of drugs can be strongly determined by the paracellular pathway. For example, certain types of endothelial cells are fenestrated (kidneys, intestines, endocrine glands) or discontinuous (liver, spleen, bone marrow) which allows the free passage of large molecules, and serves the function of facilitating drug filtration and absorption. Most other organs contain continuous endothelial cells which express junctional proteins that regulate permeability between them. The BBB has the highest expression of these proteins, including tight junction protein claudin-5, which is necessary to provide size-selective control over paracellular permeability.^82^ Only very small, nonpolar molecules like oxygen, carbon dioxide, and certain lipid-soluble substances can cross the BBB paracellularly. Larger molecules must use specific transporter systems, like glucose transporter-1 for glucose. Similarly, *in vitro* assays will be strongly dependent on paracellular permeability including gaps in monolayers of cells or differences in tight junction protein expression.^83,84^ Indeed, tight junction protein expression dramatically alters *in vitro* measurements.^83,85,86^*In vivo* and *in vitro* systems measuring permeability that is dominated by paracellular transport will be inherently inaccurate relative to *in silico* approaches, which rely on membrane diffusion. In Figure 1 we depict the transcellular pathway compared to the paracellular pathway.

In addition to discriminating between the transcellular and paracellular pathway, complex plasma membranes contain a variety of efflux pumps and transporters among an array of embedded membrane proteins. The endothelial membrane expresses P-glycoprotein (P-gp) efflux pumps (Figure 1), a notable example of ABC transporter, which are implicated in the multidrug resistance to chemotherapeutics. This pump has received notable interest since the 1980s,^87−92^ but the problem of multidrug resistance of chemotherapeutics persists.^93−95^ In the context of permeability, the P-gp pump lowers the effective drug concentration inside the cell by efflux of the drug out of the cell.

In the following sections, we lay out the theoretical frameworks for *in silico*, *in vitro* and *in vivo* permeability, specifically (A) transition-based flux counting, (B) inhomogeneous-solubility diffusion framework, (C) two-dimensional transwell assay, (D) three-dimensional microvessel assay, (E) *in situ* brain perfusion, and (F) multiple time-point regression analysis for clinical imaging. For *in silico* approaches we refer to permeability as “simulated” (*P*_sim_), while for *in vivo*/*vitro* approaches we refer to permeability as apparent (*P*_app_).

#### In Silico

**Figure 2:**
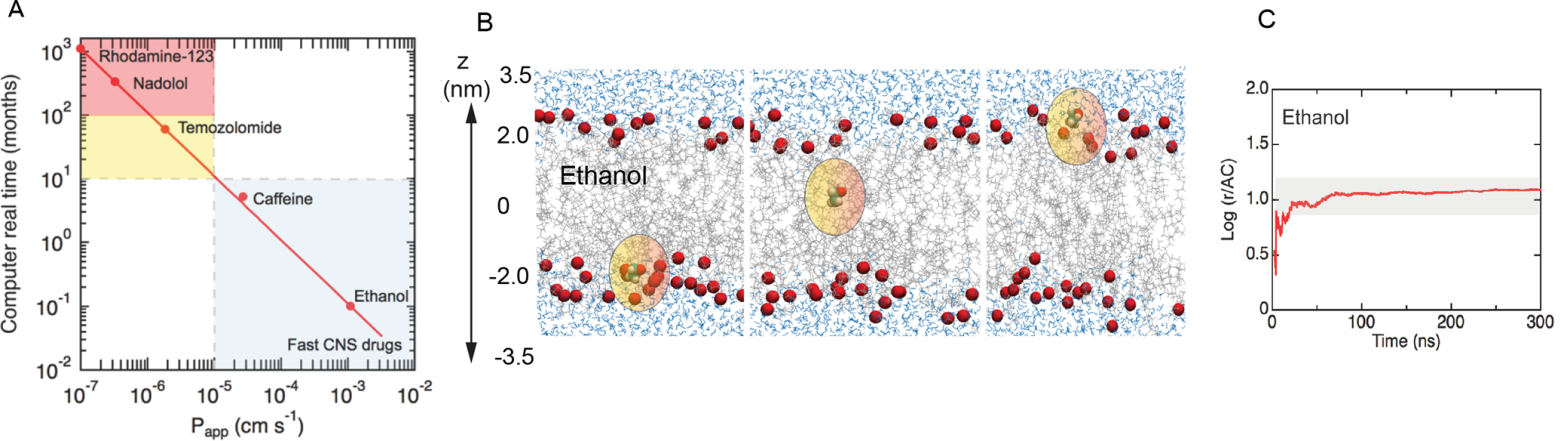
**Basics of transition-based counting approaches for permeability.** (A) Real computational time in calendar months as a function of the experimental drug permeability (*P*_app_) for the example of BBB permeation. This is obtained as a back-calculation^8^ in which the experimental permeability is input into eq 6 to calculate the expected simulation rate constant *k*, and consequently, the time *t* (*t* = *N*_event_/*k*). The time *t* refers to the time (months) to simulate solute transport at 37 °C using a modern GPU. The system setup for such a benchmark calculation is a solution volume of ∼100 nm^3^ and with a bilayer area of ∼25 nm^2^. For purposes of benchmarking, we assume that a minimum of 100 translocation events are required to achieve steady-state permeability. This is an estimate we found to provide plateau values of *k* for our compound set.^7,8,124^ A benchmark of ∼100 ns per day is assumed. The experimental values used to back-calculate to the real computational time are (Table 1) span a range from ∼10^–7^ cm s^–1^ (slowest) to ∼10^–3^ cm s^–1^ (fastest). (B) Transitions illustrated for the case of ethanol permeating across a complex membrane in an unbiased MD simulation, and for which we assume that passive diffusion to be the primary mechanism of transport. (C) Molar rate constant for ethanol throughout the MD simulation, revealing the time needed to converge rate estimates as an input to the permeability calculation. Adapted from ref (8). Copyright 2021 ACS.

Molecular Dynamics (MD) simulations utilize the numerical integration of Newton’s second law to forward-propagate the positions and velocities of atoms of an atomic representation of an assembly of atoms, thereby generating a trajectory of positions of the atoms of the system.^99−102^ The term “system” or “simulation box” here can denote any biological assembly, e.g. a cell-mimetic assembly, consisting of a model membrane, the relevant transmembrane proteins, solvent and background salt concentration. The use of MD simulations with a force field (FF) description of the solvated membrane has proven to be a popular system representation for calculating *in silico* permeability (*P*) values,^24,103^ and can be used to investigate the free energy of drug permeation across the transcellular pathway. MD simulations have been used to study a wide range of membrane types,^104−108^ including plasma and mammalian membrane models. One of the main bottlenecks in using *in silico* techniques lie in the shift between the available computational sampling (ns to μs) and the inherent biological time scales for small-molecule permeation (Figure 2). Most of the top-selling FDA approved drugs^109^ have CNS permeabilities of ∼10^–6^ cm s^–1^ and below. This renders the real-time simulation time (*t*_computing_; months) of a single GPU computationally intractable, as it corresponds to *t*_computing_ values on the order of ∼10 to 100 months for a successful calculation. To overcome this issue, the development of enhanced sampling techniques such as umbrella sampling,^110^ metadynamics,^111^ adaptive biasing force (ABF)^112^ and steered MD,^113^ have proven instrumental.^114,115^

When applying MD simulations specifically to the problem of calculating permeabilities, several procedures have been developed for use with metadynamics^116,117^ and ABF,^20,106^ among others. These methods do not directly provide transport rates without a reweighting of the resulting ensemble or inferences through an inhomogeneous-solubility diffusion (ISD) framework.^24^ Unbiased all-atom MD simulations provide detailed insights into the molecular mechanisms of transport across the BBB without the need for *a priori* knowledge of the permeation pathway or constrained coordinate systems. Kinetics can be calculated from the transition-based counting (TBC) approach, but this still requires simulation times in the tens to hundreds of microseconds per drug partition to achieve converged estimates at 37 °C. This is currently beyond the limit of realistic sampling for routine MD simulations. Other methodologies for permeability have only been parametrized for the fast regime (permeability, *P* > 10^–5^ cm s^–1^).^23^ The recent development of specialized supercomputing machines such as Anton, Anton2 and Anton3 systems has made unbiased MD simulations at the millisecond time scales routinely possible,^118−120^ but the democratized access to such machines is still not routine, and as such, enhanced sampling techniques are still required for users with access to small numbers of GPUs.

We describe two approaches to incorporate enhanced-sampling simulations, first the TBC framework, and second, the ISD framework, for transcellular permeability.

##### Transition-Based Counting (TBC)

With this methodology, the small-molecule permeability is modeled by counting the number of transport events through planes perpendicular to the bilayer located at either interface. In practice we define inward-cell and outward-cell transport planes, with the number of transport events (*N*_event_) given by the sum of the inward (*N*_i_) and outward (*N*_0_) transfers:
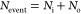
1

The rate constant, *k*, for translocation across the bilayer is calculated from unbiased MD simulations of spontaneous trans-bilayer solute crossing as the ratio of the total number of transport events observed during a simulation by the simulation time:
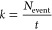
2

It is common to use a molar rate constant (*r = k*/*N*_*A*_) in calculations.

We proceed to outline the derivation for the expression of *P*_sim_. In the diffusive regime of Fick’s law, the solute flux *J* (mol cm^–2^ s^–1^) for transition events per an area patch in unit time can be expressed in terms of the simulated permeability *P*_sim_ (cm s^–1^) and the concentration difference between the two sides of the membrane:
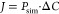
3where Δ*C* = *C*_0_ – *C*_i_, and *C*_i_ (mol cm^–3^), *C*_0_ are the concentrations on either side of the membrane. Due to the semi-isotropic pressure coupling imposed by the simulation engine with e.g. the Parrinello–Rahman barostat,^121^ in which the (XY)-plane of the box is allowed to relax independently of the (Z)-plane, the box volume, *V*, and area of the bilayer patch, *A*, will vary during the simulation and need to be averaged. *P*_sim_ is then obtained from eq 3, by estimation of the flux *J*, which itself can be obtained as the ratio of the rate *k* (s^–1^) per unit molar area (*N*_*A*_*A*; mol^–1^ cm^2^):
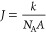
4where *N*_*A*_ is Avogadro’s constant. This leads to a permeability expression of
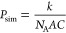
5Here *C* is the equilibrium concentration of solute in the solvent, which applies to converged simulations. Because the *in silico* permeability is calculated for a single apical membrane crossing, the factor of 2 arises to account for the both the forward and backward direction of these crossing events between the two compartments of the simulation and prevents overcounting, something which is not occurring for the supported lipid bilayers in the *in vitro* transwell assay. For this derivation, the transport across the cytosol is assumed to be much faster (*k*_cytosol_ ≫ *k*).
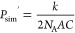
6

MD simulations allow access to transport properties as a function of the temperature of the system, such as the diffusion constant for a small-molecule. Calculation of such properties is nontrivial and is associated with a number of pitfalls explained elsewhere.^122,123^ In Figure 2 we provide an example of the workflow and time constraints for MD simulations used for permeability estimation.

Similarly, the use of temperature-enhanced MD for estimating transport properties such as the permeability^7^ is not a routine procedure and requires careful reweighting of the transport model to recover kinetics at 37 °C. Other approaches such as ABF or Umbrella Sampling require a Bayesian *posthoc* correction.^106,125,126^

##### Inhomogeneous Solubility Diffusion (ISD) Framework and Diffusion Estimation

The ISD framework (eq 7) is a popular methodology to calculate *P*_sim_ (cm s^–1^) from biased simulations, as demonstrated by Marrink et al. for water permeation.^24,127^ Uses have been shown with ABF,^106,125^ metadynamics^117^ and umbrella sampling,^128^ in which the permeability is calculated from the position-dependent diffusion *D*(*z*) and the free energy Δ*G*(*z*) along the transition coordinate *z*, and the permeability directly follows from solving the Smoluchowski equation under stationary conditions:
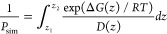
7

This approach has been widely covered elsewhere.^3,24,125,126^ One of the key challenges of these methods is the recognized difficulty to converge such calculations for heterogeneous membranes. Recently, statistical mechanical frameworks based on the Green–Kubo linear response theory have been applied to calculate *P*_sim_.^129^

One of the main requirements for using the ISD framework is a viable method to obtain the diffusion coefficients. One such approach employs Bayesian inference to obtain a consistent joint solution of *D*(*z*) and Δ*G*(*z*).^122,130^ This class of methods is compatible with any kind of bias potential, including time-dependent approaches like metadynamics^111,131,132^ or ABF.^126^ The challenge of this approach is its assumption of overdamped Brownian motion, which is not always valid, and its dependence on a time step parameter that is difficult to determine.^106^ While enhanced sampling techniques remain highly useful and extremely popular, they still require correction procedures to obtain the unbiased kinetics. The position-dependent diffusion coefficient *D*(*z*)—though less dominant in eq 7—can substantially influence the results due to the challenges in reliably estimating it.^106^

In neat solvents, diffusion coefficients can be calculated from the mean squared displacement (via the Einstein–Smoluchowski equation) or from velocity autocorrelation functions (via Green–Kubo relations). Within a lipid membrane, however, the asymmetries and large free-energy barriers preclude the approximation of the solute’s motion as a random walk, making these methods difficult to apply.^133^ Using a force autocorrelation method^134^ avoids this problem but requires constraining the solute’s position along *z*, which usually necessitates additional simulations. Since all these methods have their own advantages and disadvantages, choosing the right one usually depends on the free-energy algorithm and should be guided by optimizing the synergies between them to minimize the overall simulation effort.

#### In Vitro

Experimentally, the customary method for measuring the transcellular permeability coefficient is the *in vitro* transwell assay,^135^ which uses a cell support system in which endothelial cells are cultured to form a confluent monolayer.^29,136−138^ The role of the membrane is to support the cell layer mechanically, without acting as a significant diffusion barrier. To meet these requirements, these membranes have pores that are large enough to not restrict transport but small enough to enable cells to form monolayers (0.4 μm pores are very common). The membrane is then placed between two fluid compartments so that any flux of solutes from one compartment to the other must pass through the cell layer.

The apparent permeability (*P*_app_) of the drug is determined by measuring the amount of the molecule that crosses the monolayer.^139,140^ The molecular transport is calculated using a tracer flux assay, in which the molecular movement is tracked, which is usually done in the absence of hydrostatic or osmotic pressure gradients by having the same buffer composition and fluid height between the two chambers; thus, diffusive mechanisms will dominate and convective flux is minimal. Fluorescent compounds are commonly used for these assays as their concentration can be measured using microscopy or a plate reader, but liquid chromatography-tandem mass spectrometry can be used for nonfluorescent compounds. Many different cells are used for this assay. Many cell sources are used to model BBB permeability including Madin–Darby Canine Kidney (MDCK) or Caco-2 cells,^139,140^ primary human brain microvascular endothelial cell (BMECs),^123,124^ and BMEC-like cells derived from induced pluripotent stem cells.^141^ Dramatic permeability differences are observed across these cellular models, due to differences in tight junction protein expression,^144^ differences in epithelial versus endothelial nature of the cells, and differences in efflux pump expression.^142^

The data obtained from these experiments may then be compared to *in silico* simulations of transcellular transport to gain insights into the mechanisms of BBB permeability.^3,20−26^ However, the choice of cell types and experimental conditions can significantly influence assay outcomes, underscoring the need for standardized methodologies and quality control measures. The work of Wong et al.^29^ succinctly summarizes the experimental procedure. We start the derivation from Fick’s law of diffusion (eq 8), which governs the solute flux. Let *J* denote the solute flux, expressed in units of mol m^–2^ s^–1^, *D* denotes the diffusion constant of the solute, which is dependent on intrinsic (i.e., size of solute) and extrinsic (i.e., temperature) parameters, and is expressed in units of m^2^ s^–1^. Let d*C*/d*x* denote the concentration gradient in ((mol m^–3^)/m). In Fick’s Law, the minus sign indicates that solute is transported in the direction of decreasing concentration:
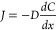
8

This calculation can be simplified to define the flux (*J*) in units mol cm^–2^ s^–1^ across a permeable membrane by assuming steady-state diffusion (constant concentration gradient) and that the membrane is thin (no complex gradient). *P* is the permeability coefficient (cm s^–1^), and Δ*C* is the concentration gradient (mol cm^–3^).
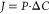
9

##### Two-Dimensional Transwell Assay

**Figure 3:**
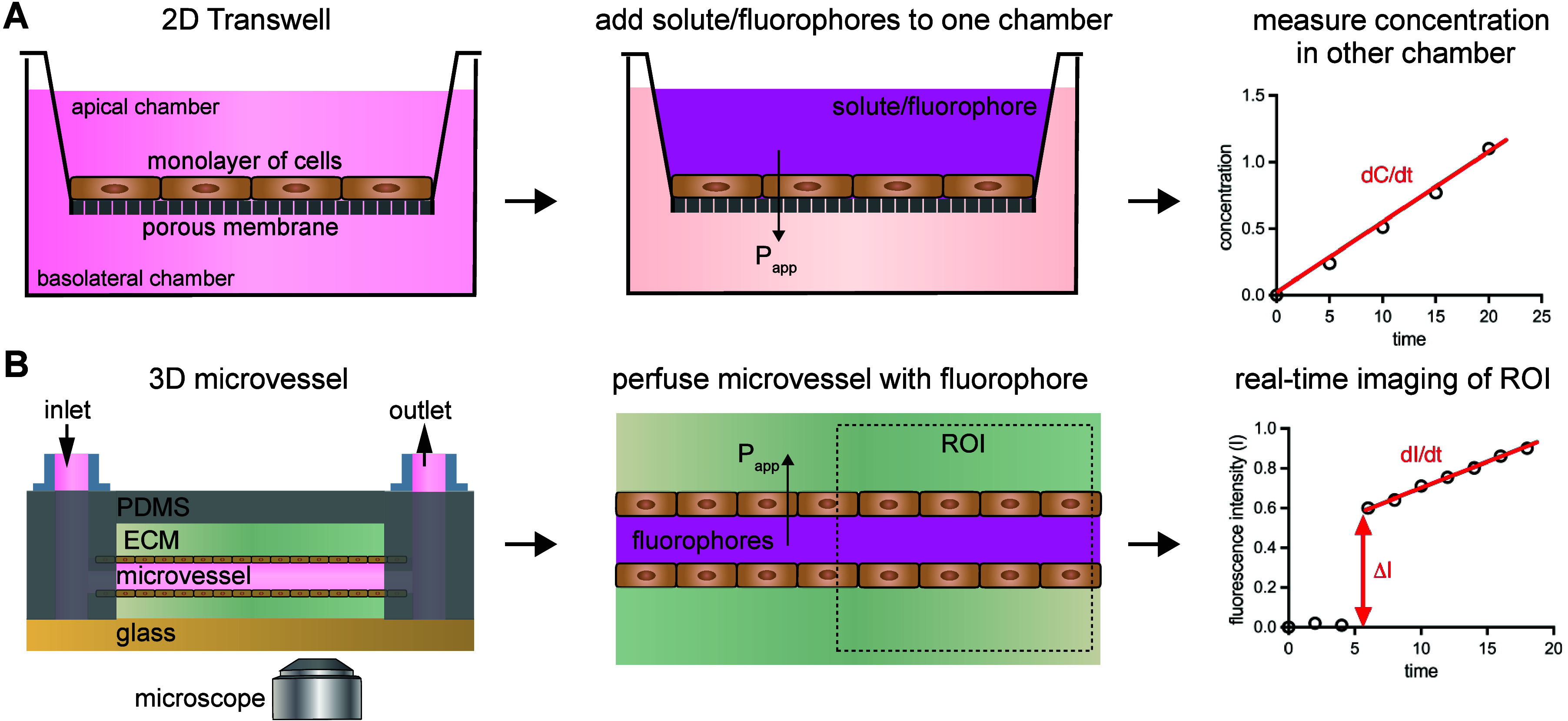
*In vitro***measurements of permeability.** (A) Schematic of 2D Transwell assay for measuring permeability of cell monolayers. Cells are grown on a porous membrane; if a monolayer does not form then transport between cells (paracellular) will dominate, which may not reflect the physiological barrier properties. Solute or fluorophore is added to one chamber and the concentration over time is measured in the opposing chamber. Measurements will only be accurate in the linear regime of d*C*/d*t*. Typically, a standard curve is used to calculate the concentration of a fluorophore and its change over time in the basolateral chamber using a plate reader. From this curve, permeability is calculated by eq 13, which also is dependent on the geometry of the Transwell membrane (typically circular). (B) Schematic of a 3D microvessel assay for measuring permeability of cells patterned into a tube. Tissue-engineering approaches are used to form a confluent tube of cells (typically endothelial cells) that resembles a *in vivo* blood vessel. Fluorophore is perfused through the microvessel and real-time fluorescence microscopy is conducted to capture filling of the lumen (Δ*I*) and the rate of fluorophore accumulation into the ECM (d*I*/d*t*), together with geometric properties of the microvessel enabling permeability calculation (eq 17). In the example plot of fluorescence intensity, the jump in fluorescence corresponds with perfusion of dye into the microvessel at time = 5 min. This calculation does not depend on measuring the concentration of the fluorophore, however, a range of concentrations should be piloted to confirm linearity with fluorescence and robustness of measurements. Polydimethylsiloxane (PDMS); extracellular matrix (ECM).

A Transwell forms a permeable membrane containing a cell monolayer with area *A* that is separated by an apical and basolateral chamber with volumes of *V*_apical_ and *V*_basolateral_, respectively, as depicted in Figure 3A. To measure permeability, a compound of known concentration (*C*) is placed in a chamber to establish a concentration gradient. Typically, this is done by replacing media from the apical chamber. After media is replaced, solute will transport into the basolateral chamber eventually reaching a plateau based on the differences in volume between the chambers (it will plateau at 50% of initial concentration if the reservoirs are the same volume). In practice, this means that measurements should be collected in the linear regime of transport to satisfy steady-state assumptions. To identify the linear regime, time course studies are usually conducted to identify when flux starts to plateau (this occurs much more rapidly for high permeability solutes). Over time, fluids in the basolateral chamber are collected and replaced with fresh buffer to re-establish a concentration gradient and to measure the concentration over time.

The flux for the transwell assay is defined as the rate of molecules crossing the membrane (d*N*/d*t*) divided by the surface area:
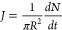
10which is the form of eq 4 for a circular area. Assuming that the solute is only present in the apical reservoir, the concentration gradient d*C* for the transwell assay is defined as
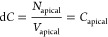
11

Thus, apparent permeability is defined from eq 9 as
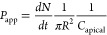
12

By redefining the permeability in terms of the solute concentration, we get:
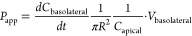
13

The first term can be calculated as the slope of concentration across at least two time points of sampling the basolateral compartment.

##### Three-Dimensional Microvessel Assay

A three-dimensional microvessel is fabricated using cell and tissue engineering techniques, summarized previously.^143^ The microvessel of length *L* and radius *R* is filled with solute of amount *N*_vessel_, and over time outside of the blood vessel this solute accumulates at rate d*N*/d*t*. The calculations of permeability are similar to those in 2D, and the final experimental setup is depicted in Figure 3B. First, the molecular flux is similar to the 2D expressions (eqs 9–11):
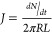
14
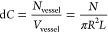
15

Solving for permeability, the expression then becomes
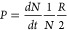
16

Typically in these models, permeability of fluorophores are measured. Unlike in transwells, where the basolateral chamber is accessible for fluid collection, in microvessels there is not a tractable way to sample the interstitial space. Thus, time-lapse fluorescence microscopy is typically conducted to measure the concentration gradient when the microvessel lumen fills with fluorophore (Δ*I*) and how fluorescence intensity changes over time outside the microvessel (d*I*/d*t*). These equations were applied in early studies to measure the permeability of single perfused capillaries of frog mesentery,^144^ but have been widely adopted within the *in vitro* microvessel field.^137,145,146^
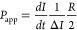
17

These calculations make several assumptions: (1) fluorescence intensity is linear with concentration, which should be empirically confirmed, (2) microvessels instantaneously fill with fluorophore (immediate formation of a concentration gradient); if this is not satisfied then transport will occur before the final concentration gradient is formed; using a perfusion system that has a long physical distance from the site of adding fluorescent dyes to the microvessel can introduce this challenge, which will particularly effect high permeability measurements, (3) the concentration gradient is steady-state, (4) that all transport occurs within the ROI, meaning that the ROI should be chosen to reflect transport from the singular microvessel, and (5) that diffusion dominates, while contributions of convective flux (solvent drag) are minimal. Convective flux is described by the Sterling equation and includes both hydrostatic and oncotic pressures. Hydrostatic contributions can be minimized by perfusing microvessels under low pressure, while oncotic effects can be minimized by ensuring that the interstitial fluid surrounding microvessels has a similar protein concentration as the microvessel perfusate. For example, perfusion of high concentration fluorescently labeled albumin will introduce oncotic effects that will draw water into the microvessel and alter permeability beyond diffusive mechanisms.

#### In Vivo

Historic studies by Ehrlich and Goldmann found that dyes injected into the bloodstream did not stain the brain,^147^ providing early quantitative evidence of uniquely low permeability of the BBB. Quantitative and semiqualitative methods continue to be used to this day using dyes like Evans Blue or biotin to observe how barrier properties change over development and disease. Regardless, there remains a need for accurate quantitative measurements and multiple techniques have been developed to measure permeability in living animals or humans (*in vivo*).^148−150^ These approaches are applied across broad orders of magnitude of size, from the collection of entire organs to measure drug concentration to the real-time imaging of a single blood vessel perfused with solute directly upstream. Calculations across these scales differ widely and rely on unique sets of assumptions that can bias measurements. Some early approaches perfused solutes through animal microvessels and utilized calculations outlined above (eq 17);^143,145^ these approaches have also been applied with more advanced imaging modalities and noninvasive techniques to measure BBB permeability.^151^ However, there remains a need for measuring permeability that does not rely on the direct fluorescent imaging of a vascular bed. We will describe two such approaches: (1) *in situ* brain perfusion which is a “gold standard” approach to measure permeability of any drug in laboratory animals, and (2) multiple time-point regression analysis, including the two-comportment Patlak model, which is widely used for clinical imaging in humans.

##### *In Situ* Brain Perfusion

In this method the operator takes full control of the cerebral circulation by isolating and cannulating the internal carotid artery in a freshly euthanized animal and perfusing with a solution of tracer of known concentration (*C*_*p*_). The concentration of tracer in the brain (*C*_*b*_) is quantified, either at a single time point or at multiple time points. For multiple time points, the initial linear part of the data is then fitted to this relationship:
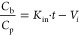
18where *C*_*b*_/*C*_*p*_ is the apparent volume of distribution, the slope *K*_in_ is the transfer coefficient (expressed in mL/min/gram of brain tissue) and the intercept *V*_i_ (at *t* = 0) reflects the intravascular volume. For single point assays, *V*_i_ needs to be determined empirically and this is done by including in the perfusate an intravascular space marker which has zero BBB permeability. Multiple time points are superior to single time point assays since it allows for inspection of the relationship in eq 18, to ensure the calculation is performed on the linear portion.

The determinants of *K*_in_ are the permeability (*P*_*,*_ an intrinsic property of the BBB; when measured referred to as *P*_app_ versus *P*_sim_), the surface area available for transfer (*S*), and luminal flow (*F*). *P* and *S* are convenient to consider together as a product (so-called “PS product”, or *PS*), since it is frequently difficult to disentangle these two parameters:
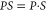
19

*PS* was related to *K*_*in*_ and *F* by Renkin^152^ and Crone,^153^ so *K*_in_ can be derived since the flow is controlled by the experimenter, and is known or can be derived:
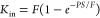
20rearranged to
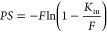
21but in practice some investigators assume that *K*_in_ is numerically equal to *PS* when flow is much larger than the *PS* product (simulations show that when *PS/F* is less than 0.2, *K*_in_ approximates to *PS* with error ≤10%^154^).

Finally, *P* (in cm/min) is derived from *PS* by dividing with estimates of vascular surface area (*SA*, in cm^2^/g) from the literature,^155^ since from eq 19:
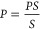
22

These experiments are essentially performed post-mortem and the brain is deprived of its normal blood supply. While animals are perfused with a highly oxygenated and warmed solution (sometimes including washed erythrocytes), it is possible that BBB permeability changes occur during the course of the experiment as the neurovascular unit is deprived of its natural environment.

##### Multiple Time-Point Regression Analysis

A more physiological extension developed by Patlak and colleagues allows for permeability estimation after administration of tracer. For CNS permeability, the procedure involves obtaining concentrations measured in tissue (*C*_b_) and blood (usually arterial, so *C*_a_) at multiple time points.^155^ The Patlak model is used in laboratory studies and clinical imaging techniques like PET (Positron Emission Tomography) and dynamic contrast-enhanced magnetic resonance imaging (DCE-MRI);^156−158^ it measures the rate at which an agent (dependent on the imaging modality) moves from the blood plasma (compartment one) into the tissue (compartment two). Crucially, this is a two-compartment model which assumes that tracer is irreversibly transferred from blood to tissue (i.e., no back flow).
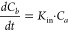
23since at early time-points, the influence of *K*_out_ · *C*_a_ is very small, as there is very little tracer in the tissue compared to blood.

Integration of eq 23 leads to
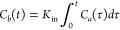
24

To the right-hand side of eq 24, one needs to add the contribution of intravascular tracer to *C*_b_ i.e. *V*_b_. *C*_a_(*t*) where *V*_b_ is the cerebral blood volume. This is the Patlak equation:
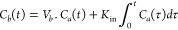
25

Dividing by *C*_a_(*t*) purely to rearrange into a linear form:
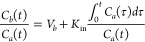
26allows the data to be plotted to derive *K*_in_ and *V*_b_ by fitting. The advantage of this method is that tissue concentrations can be measured *in vivo* using imaging techniques, and application to humans becomes possible.^159^ Further improvements have been made using bidirectional models,^160,161^ adjustments for noninstantaneous luminal filling,^162^ and “blind” deconvolution of solute imaging data.^157,163−165^ Depending on the context, different adaptations of the Patlak model can be more or less accurate. For example, bidirectional models are more accurate when vascular permeability and vascular volume are high.^157^ As with other approaches, this model assumes that the signal is proportional to concentration, and is popular with a variety of clinical imaging modalities;^155,166,167^ depending on the imaging sequence, tissue characteristics, and contrast agent and its concentration, a linear relationship can be approximated. *K*_in_ is commonly also referred to as *K*_i_ (or *K*_trans_, if *K*_i_ is corrected for the hematocrit) in MR perfusion studies, such that
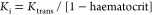
27

By constraining eq 20 within the physiological ranges of *F* and *PS* across various tissues, it is apparent that *K*_in_ is mostly influenced by *PS* (luminal flow (*F*) has little effect), especially when the *PS* is very low. Indeed, in the low permeability context, *K*_in_ is nearly directly proportional to *PS*.

### Challenges in Benchmarking Permeability Measurements

#### Quantifying the Order of Magnitude Difference between Simulation and Experiments

Directly comparing simulated and experimental permeabilities involves inherent risks. Simulated permeabilities for the CNS have been found to generally be faster than experimental permeabilities even after correction procedures. This has been found for simulated permeabilities obtained with transition-based counting assays^7,8^ that are then benchmarked to Caco-2, MDCK or rat brain perfusion. Similarly, for simulated permeability values obtained from the ISD relationship, benchmarking to *in vivo* intestinal perfusion assays revealed a disparity of 3–4 orders of magnitude,^168,169^ with the simulated values being significantly faster. The reason for this has been extensively explored,^8,124,169^ and appears to be a combination of force field effects, the uniqueness of each permeating solute in relation to a single assay that may not respond to all solutes in the same way, as well as the choice of *in vitro* cell line benchmark or *in vivo* methodology with their inherent limitations. We will delve more deeply into these issues in the following subsections.

#### Building Atomic-Detail Accurate Models

One key reason for the order-of-magnitude disparity between simulations and experiments is that small-molecule tissue permeability through a cell membrane is affected by a multitude of biological factors within *in vivo* and *in vitro* models (Figure 1). These factors are only captured by the simulation model if the corresponding features are explicitly included. First, brain microvascular endothelial cells (BMECs) are interconnected by tight junction proteins that form a paracellular barrier to the transport of large molecules. By electron microscopy these proteins appear as “zippers”.^170−172^ Thus, experimental and computational approaches would be expected to converge for blood–brain barrier permeability measurements when paracellular permeability goes to zero. Second, the endothelial cell membrane is coated in a layer of glycan molecules termed the glycocalyx, which helps preorganize the transport for small molecules and blocks the permeation of larger molecules.^96,98,173^ Since simulation models do not routinely include the glycan layer in their modeling, this is a major system difference. The permeability for large molecules when benchmarked to *in silico* permeabilities therefore needs to be scrutinized extra carefully, and this discrepancy could be mitigated by stripping the glycocalyx from the cell surface prior to permeability measurements using compounds like heparinase.

Third, simulation models do not routinely incorporate P-glycoprotein efflux, which can lead to order-of-magnitude differences in *in vitro* permeability.^174−176^ To quantify the effect of efflux on the permeability, we quote the concept of “efflux ratio” (eq 28) to denote the ratio of the basolateral-to-apical (brain to circulatory direction) to the apical-to-basolateral (circulatory to brain) directions:
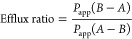
28

By rewriting the total system flux *J* from eq 3 in terms of passive and active transport components, we obtain expressions for both components to be
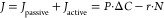
29where *r* is the transporter turnover rate and *N* is the number of transporters. The consequence of active transport is that the total flux may no longer be linearly dependent on the concentration difference, depending on the rate of active transport. Furthermore, when evaluating eq 29, it may help to point out that *N* is typically found as the number of transporters per micron squared.^29,177,178^ In the case of the P-glycoprotein transporter, a value of 100 P-gp μm^–2^ has been used previously.

Fourth, the cell membrane composition *in vivo* is dynamic and regulates other mechanisms of transcellular permeability like transcytosis. Relative to other vascular beds, the BBB is enriched for the transporter protein Mfsd2a^179^ which regulates the lipid composition of the cell membrane to suppress caveolae-mediated transcytosis.^180^ Fifth, many compounds use specialized transporter systems to cross the BBB, which dramatically increase permeability of the transcellular pathway; this includes expression of glucose transpoter-1 by the BBB to shuttle glucose into the brain. To capture such effects *in silico*, we are thus advocating for users to move on from model membranes to complex simulation setups, such as those mimicking the full cell,^181^ but this comes at a considerable computational cost and is not routine.

#### Assumptions of in Vivo Permeability Estimation

Having gone over differences between the simulations and the reality of the cell *in vivo,* we proceed to examine the assumptions made for *in vivo* permeability estimation of *P*, the gold standard in CNS permeability estimation. For CNS permeability estimation, one assumes that minimum binding occurs on the luminal surface of the vasculature, as well as that there is no additional barriers in the perivascular space; although the permeability in relation to the cerebrovascular endothelium would be mathematically correct, it would not be accurate for a systems biological application, since most therapeutics and biological substances need access to the brain parenchyma to exert their action. Transfer kinetics are assumed to follow a one-compartment unidirectional model; multicompartmental models of increasing complexity^182,183^ including those utilizing measurements from extracellular space and cerebrospinal fluid compartments attempt to deal with this issue,^184,185^ for which there is a risk of overfitting with large number of parameters. Absolute quantification of *P* is hampered by the fact that the surface available for transfer, denoted *S*, is derived from the literature, rather than being experimentally determined within the same experiment. However, since *S* is essentially a scaling variable, it is acceptable to compare permeabilities between substances (e.g., *P*_app, substance 1_ and *P*_app, substance 2_), or to compare *P*_app_ and *P*_sim_ if a reference substance is used (e.g., comparing *P*_app, substance 1_/*P*_app, ref_ and *P*_sim, substance 1_/*P*_sim, reference substance_).

#### Diversity of Solute Permeability Behavior

In addition to these systemic differences between simulations and experiments, there is a diversity in the behavior and nature of the small molecules studied, which need to be accounted for in benchmarks and models. We illustrate this issue for three classes of molecules crossing the BBB. The first type of compound is one with moderate to high permeability across lipid membranes. Both caffeine (2 × 10^–5^ cm s^–1^)^8^ and ethanol (1.1 × 10^–3^ cm s^–1^) serve as examples for such behavior (Table 1), and both immediately exert noticeable physiological effects on the CNS after their respective consumption. A second class of molecules with moderate to low permeabilities are the opioids, that have highly opioid-drug specific entry into the brain. Some opioids are strongly recognized by P-glycoprotein, such as loperamide^186,187^ (which exerts no CNS actions), while others such as heroin or morphine are effluxed to a lesser extent.^188,189^ A third class of molecules are the very large drugs such as antibodies. Antibodies display remarkably low permeability unless they are engineered to hijack receptors expressed by the brain endothelium, including transferrin receptor.^190^ To study the permeability of large molecules, novel delivery platforms such as the transcytosis-enabling modules (TEMs)^191,192^ are needed. In Table 1 we illustrate a range of compound permeabilities for the blood–brain barrier, showcasing the diverse set of assay types used to estimate these. Future benchmarks need to be aware of this diversity and the challenges it poses to accurate assessment.

#### In Vitro Models Have Inherent Advantages and Limitations

*In vitro* permeability assays have a set of common pitfalls, recently described elsewhere.^193^ To summarize, first, only a short-range of *P*_app_ values allow for the extraction of intrinsic membrane permeability, as most of the published values are dominated by the diffusion component, which itself is due to unstirred water layers.^169^ Second, it was found that many of the non-CNS *P*_app_ values are affected by paracellular transport when using epithelial or intestinal permeability assays. This is not an issue in CNS permeability, due to the presence of tight junctions that block paracellular transport. Third, there are recovery issues in the *in vitro* assays, which are not present in the simulations, as compounds are lost due to adhesion to membranes or plastics.

#### Benchmarking Disparities between in Vitro and in Vivo

*In vitro* systems do not fully recapitulate the complex nature of drug delivery *in vivo*, e.g. in humans. This includes differences in protein/gene expression of endothelial cells, microenvironmental differences, and incomplete capture of ADME principles *in vitro*. As a first example, let us consider the permeability of clozapine, an antipsychotic drug. In Table 2 we compare the measured permeability for the antipsychotic clozapine from three sources. While the experimental sources differ in their assumptions, the fastest permeability reading was 6.4 times greater than the slowest reading. *In vivo* methods measure molecules crossing the membrane by all mechanisms. The permeability is therefore not driven by passive diffusion or unequal concentrations alone. If the delivery depends on an active transport system, the kinetics will look different than transport that is just following a gradient, particularly if the concentrations are very high.

**Table 2:** Experimental Benchmarks for the Apparent Permeability of the Antipsychotic Clozapine from the Following Models or Methods: (A) 2D *in Vitro* Permeability from Human Cerebral Microvascular Endothelial Cell Line (hCMEC/D3) Monoculture Cells, (B) 2D *in Vitro* Permeability from Madin-Darby Canine Kidney Cells with Multidrug Resistance Protein 1 Expressed (MDCK-MDR1), and (C) 3D *in Vivo* Permeability from Rat Brain Perfusion

Molecule	MW(g mol^–1^)	Log *K*_ow_	*P*_app_ (cm s^–1^)	*P*_app_ Reference	*In vitro* model or *in vivo* method
**Clozapine**	326.8	3.23	3.90 × 10^–5^	[Yang 2024]^68^	hCMECD/D3
			2.80 × 10^–5^	[Summerfield 2007]^68^	MDCK-MDR1
			2.50 × 10^–4^	[Summerfield 2007]^68^	Brain perf (3D)

Second, a recent study^194^ collected 222 *P*_app_ values from MDCK cell lines, and 143 *P*_app_ values from Caco-2 cell lines. When analyzing the *P*_app_ values for the same chemical, it was found the *P*_app_ to differ by up to ∼1.83 log units with a median of 0.57 when collected from different sources. When analyzing *P*_app_ values of the same chemical from the same source where only the choice of cell line was different, the values differed by up to ∼1.31 log units with a median value of 0.31. This highlights the choice of the cell line as having an impact on benchmarking. The observed differences in permeability between Caco-2 and MDCK cells are primarily attributed to variations in lipid and protein composition, as well as cellular morphology, with Caco-2 cells more closely mimicking the human intestinal barrier while MDCK cells resemble renal epithelium; these distinctions underscore the importance of cell line selection in accurately modeling permeability.^195^

Third, *in vitro* studies commonly cite *in vivo* measurements as a reference point for permeability values. This is typically used as evidence for an *in vitro* model displaying “physiological” barrier properties. Unfortunately, this practice has large potential for error as there is not an accepted *in vivo* value for many compounds as they are highly dependent on the approach used. Pairs of *in vitro* and *in vivo* studies identify similar permeability values, but values are still highly variable across approaches; examples of congruent permeability findings for two compounds include for lucifer yellow^137,196^ and 10 kDa dextran.^197,198^

Fourth, *in vitro* methods rely on spectroscopy to detect fluorescence intensity. Some limitations that may compromise accurate measurements include nonspecific binding of fluorophores to Transwells, photobleaching of the fluorophore, interference or autofluorescence of cell culture media (thus why transport buffers are usually used during the assay), and instrument sensitivity.

#### Measuring Modulation in Permeability

Vascular permeability is highly dynamic in homeostasis and disease. Additionally, many approaches seek to actively increase permeability to delivery therapeutic agents into the brain. There is a large range of approaches utilized including inhibition of efflux pumps, transient disruption of tight junctions (by hyperosmotic/chemical agents, focused ultrasound), hijacking of receptor-mediated transcytosis, and use of viral vectors, nanoparticles, or exosomes.^199,200^*In silico, in vitro*, and *in vivo* approaches have all been applied toward developing approaches to modulate drug permeability. However, some benchmarking challenges persist. For example, due to species differences it is possible that permeability measurements may be distinct between *in vivo* studies in rodents versus studies using human cells *in vitro*; the relative contribution of species differences versus technical artifacts is not known but could at least partially be determined by conducting gene and protein expression analysis across these assays. Crucially, while assumptions may be satisfied in one context, once permeability is modulated they may no longer be valid. For example, many chemicals can induce “focal leaks” within 3D *in vitro* models where plums of fluorophore are observed to transiently exit between adjacent endothelial cells.^145,201^ These transient effects are local sites of extremely high paracellular permeability and suggest that the ratio of transcellular:paracellular permeability is not constant in time or space. Permeability may be underestimated in the presence of focal leaks as the concentration gradient is poorly maintained. Despite these challenges, this highlights a major advantage of 3D models: the direct imaging of fluorophore dynamics, which is not visible in 2D models where fluid is passively collected from reservoirs.

### Guideline Recommendations for Improving Permeability Assay Design

The previous section discussed the challenges on benchmarking, with the ultimate goal to improving agreement between *in vitro*, *in vivo* and *in silico* prediction. The advantage of this is that (i) by achieving congruent measurements between *in vitro* and *in silico* systems we enhance the accuracy of such methods, (ii) by achieving congruent measurements between *in vitro* and *in vivo* systems we enable the use of *in vitro* systems to study permeability dynamics without the use of animal models, and (iii) by achieving congruent measurements between *in silico* and *in vivo* we demonstrate the translatability of these measurements to predict drug permeability.

We now present a community-led assessment of the best practices to follow for improving permeability assay design:

1. *Work out the explicit assay assumptions prior to performing a benchmark*. During large data set benchmarking between simulated permeabilities and experiments (Table 1), one should be considerate of the specific details and opt for choices that *improve* the overlap of experimental conditions. These choices include the cell line, the incorporation or inhibition of efflux effects in the assay, and the type of solute being benchmarked. A justification needs to be made in relation to their potential weaknesses. The choice of the cell line can have order-of-magnitude differences on the experimental permeability, while efflux effects are known to have significant impacts on the permeability,^29^ and this needs to be carefully accounted for in assay design, such as opting away from MDCK-MDR1 transfected cells, in which P-gp effects are captured, or correcting for this in the transport coefficients.
2. *Nature of permeant affects outcome.* As we discussed above, there is ample evidence that solutes permeate complex membranes in group-type behavior.^8,202^ Where atypical sized solutes are utilized, consider the challenges of such a class of solute. Brocke et al. proposed five classes of permeant behavior through complex membranes from simulations,^202^ while other groups narrowed this down to three discrete classes of behavior.^8^ Similarly, peptide permeability requires specialized assays that differ from those of small-molecule permeability, and do not generally follow the Lipinski rule of 5.
3. *Data utilization and data availability*. To improve the transparency of benchmarking, we recommend following the FAIR (Findability, Accessibility, Interoperability, and Reusability) data depositing guidelines recently proposed,^203,204^ as this will improve the agreement to future simulation setups and help new users in the community follow best practices. In practice, this means depositing raw publishable data in a repository in such a manner that the data arrangement obeys the FAIR guidelines. We do not have a single repository in mind, as we appreciate the scientific community is global and has unique challenges in different parts of the world, but some popular ones include MDverse,^205^ MDDB (https://mddbr.eu/) and BioExcel-CV19 (https://bioexcel-cv19.bsc.es/). We recommend users follow the best practices for data sharing, such as those laid out by JCIM.^206^ Similar efforts for experimental data deposits have been proposed, e.g. NCBI GEO databases to deposit gene expression and epigenomics data sets generated by next-generation sequencing.^207^
4. *Data checking.* We recommend that users carefully check the input data used for *in silico* prediction. This includes standard error checking to ensure the best practices are followed. As an example, the ISD framework requires inputs from both thermodynamics and diffusion estimation. A breakdown of the error analysis and uncertainties for thermodynamics^108,208^ and diffusion^122,123,209,210^ have been reported elsewhere. Following good practices^208^ for calculating thermodynamics is of utmost importance to ensure reliable data. For the TBC approach, the monitoring of the convergence and rigorous derivation of the permeability expression has been discussed elsewhere.^7,24^ Similarly, the use of simulation force field for small-molecules, e.g. GAFF^211^ or CGenFF,^212^ can vary widely in accuracy, and careful reoptimization or inspection of the associated assigned penalties is very important.
5. *Avoid pitfalls*. For *in vitro* measurements, it is advised to confirm integrity of cellular monolayers prior to measurements to ensure that transport is not dominated by the paracellular pathway. This can be done using transendothelial electrical resistance (TEER) measurements and values can be benchmarked to prior studies for consistency.^29^ Second, make sure to measure transport in the linear regime, by conducting time course studies.^88^ Third, make sure fluorescent intensity is linear with concentration, which has been a standard practice for decades.^144^ Lastly, test the assumption validities in your experimental or simulation setup. A number of resources for good experimental^213−216^ and simulation^204,208,217^ setups (or “best practices”) exists, and the readers are directed to those.
6. *Data replication.* Because of the complications of attaining agreement between experimental and simulated permeability, it is paramount that values be given with statistical error bars, obtained from repeat experiments or simulations. An excellent reference framework for this recommendation is the work of Summerfield et al.^66^ in which permeabilities from at least two methodologies are reproduced side-by-side descriptors of mean and error.
7. *Embrace multidisciplinary efforts.* Use of multidisciplinary efforts will be particularly powerful in advancing studies of permeability. Given inherent assumptions and limitations in individual approaches, collaborative efforts to benchmark and compare across experiments and simulations will strengthen confidence in findings and hint at mechanisms. For example, *in vitro* and *in vivo* work recently applied the bee venom peptide melittin for transient opening of the BBB.^218^ Use of both an *in vitro* model using human cells and rodent models, are complementary preclinical studies that hint at dose discrepancies between *in vitro* and *in vivo* that require further study. While this work speculates on mechanisms by live-cell imaging of 3D microvessels, recent *in silico* studies of melittin identify the membrane permeabilization mechanism.^219^ This example highlights the potential for *in silico, in vitro*, and *in vivo* approaches to synergize toward improving drug delivery.

## Conclusion

In this community assessment, we have laid out the analytical framework for multidisciplinary methodologies to calculate permeability: *in silico* assays using either transition-based counting or the inhomogeneous-solubility diffusion approaches, *in vitro* permeability assays in 2D or 3D, and *in vivo* assays utilizing *in situ* brain perfusion and the Patlak method for clinical imaging. We have gone through the systematic derivation of permeability expressions, and covered the underlying assumptions made for each approach. We have performed a systematic benchmarking of *in silico* to both *in vitro* and *in vivo*, depicting the ways in which each benchmarking is sensitive to the choices of assay design. Finally, we outlined recommendations for best practices in permeability benchmarking and underscore the significance of tailored permeability assays in driving advancements in drug delivery research and development across diverse physiological contexts. During benchmarking between simulated permeabilities and experiments, the following should be taken into consideration: (1) assay variability, including cell line and efflux effects; (2) the class of solutes; (3) data utilization and data availability; (4) input data checking for the *in silico* prediction; (5) avoiding pitfalls; (6) data replication; and (7) embracing multidisciplinary efforts.

## Data Availability

The data in Table 1, as well as rudimentary scripts to evaluate the transcellular molecular rate constant, are to be found at the repository https://github.com/chrisjorg/Permeability.

## Abbreviations

CNS: central nervous systems
BBB: blood–brain barrier
FDA: Food and Drug Administration
PBPK: physiologically based pharmacokinetic
ToF-SIMS: Time-of-Flight Secondary Ion Mass Spectrometry
MDCK: Madin-Darby canine kidney
Caco2: immortalized cell line of human colorectal adenocarcinoma cells
hCMED/D3: Human Cerebral Microvascular Endothelial Cell Line monoculture cells
MDCK-MDR1: Madin-Darby canine kidney with Multidrug Resistance Protein 1 expressed
hBMEC: human brain microvascular endothelial cell
iPSC: induced pluripotent stem cell
ISD: inhomogeneous solubility-diffusion
ABF: adaptive biasing force
2D: two-dimensional
3D: three-dimensional
